# A gene-rich and compact chloroplast genome of the green alga *Nephroselmis pyriformis* (N.Carter) Ettl 1982 from the shores of Mersin (Eastern Mediterranean Sea)

**DOI:** 10.1080/23802359.2020.1866461

**Published:** 2021-02-05

**Authors:** Romain Gastineau, Merve Konucu, Dilek Tekdal, Claude Lemieux, Monique Turmel, Andrzej Witkowski, Elif Eker-Develi

**Affiliations:** a Institute of Marine and Environmental Sciences, University of Szczecin, Szczecin, Poland; b Department of Biotechnology, Mersin University, Institute of Graduate Studies in Science, Yenişehir, Mersin, Turkey; c Department of Green Chemistry and Technology, Ghent University BW24, Gent, Belgium; d Faculty of Science and Letters, Department of Biotechnology, Mersin University, Yenişehir, Mersin, Turkey; e Département de biochimie, de microbiologie et de bio-informatique, Institut de Biologie Intégrative et des Systèmes, Université Laval, Québec, Canada; f Faculty of Education, Department of Mathematics and Science Education, Mersin University, Ciftlikkoy, Mersin, Turkey

**Keywords:** Nephroselmis, chloroplast genome, Eastern Mediterranean Sea, gene-rich and compact, chlorophyta

## Abstract

We report the complete chloroplast genome of the MED1 strain of *Nephroselmis pyriformis* from the Eastern Mediterranean Sea. At 111,026 bp, this genome is smaller and more compact than those of *Nephroselmis olivacea* and *Nephroselmis astigmatica*, and in contrast to the latter taxa, its inverted repeat contains no complete protein-coding genes. It encodes 3 rRNAs, 33 tRNAs and 94 proteins. Maximum likelihood analysis of a concatenated set of chloroplast genes from green algae belonging to deep-diverging lineages positioned the three *Nephroselmis* species in a strongly supported clade in which *N. pyriformis* is sister to *N. astigmatica*.

Representing a basal lineage of the Chlorophyta (Nephroselmidophyceae), the green algal genus *Nephroselmis* comprises 13 taxonomically accepted species of unicellular flagellates with a reniform shape and two unequal flagella (Leliaert et al. 2016; Guiry and Guiry 2020). Except for *N. olivacea*, which is freshwater, all *Nephroselmis* species inhabit marine or brackish waters. *Nephroselmis pyriformis* (N. Carter) Ettl 1982 (etymologically, ‘pear-shape’) can be distinguished based on the number and shape of its body scales (Yamaguchi et al. 2011). Known as a cosmopolitan species, *N. pyriformis* was first observed in the Eastern Mediterranean Sea in 2015 (Eker-Develi 2015). This identification, which was based on light microscopy, was later confirmed by Scanning Electron Microscopy and molecular barcoding of its partial SSU gene (GenBank: MN559709) (Konucu et al., 2019). A separate strain of *N. pyriformis*, strain MED1, was isolated in February 2016 from samples of the Eastern Mediterranean Sea taken near Erdemli (Turkey) (36°36′N, 34°19′E). In the present study, we report the chloroplast genome sequence of this strain and compare it with the genomes of the two other *Nephroselmis* species that are currently available in public databases: *N. olivacea* (GenBank: AF137379, 200,799 bp) (Turmel et al. 1999) and *N. astigmatica* (GenBank: KJ746600, 125,042 bp) (Lemieux et al. 2014).

The *N. pyriformis* MED1 strain, together with frozen biomass and DNA samples of this strain, are being kept in the Department of Biotechnology at the University of Mersin. DNA sequencing was performed by the Beijing Genomics Institute (Shenzhen, China) on the DNBSEQ platform. A total of *ca*. 40 million 150-bp paired-end reads were generated and assembled using SPAdes 3.14.0 (Bankevich et al. 2012). Following the identification of chloroplast contigs, the chloroplast genome sequence was completed using Consed (Gordon and Green 2013). Genes were identified as previously described (Turmel et al. 2017).

The *N. pyriformis* genome (GenBank: MW077730) contains 128 conserved genes that encode 91 proteins, 3 rRNAs, 33 tRNAs and a *rnpB* gene, and also displays 3 additional open reading frames (120, 160 and 186 amino-acid long). At 111,026 bp, it is smaller and more compact than the *N. olivacea* (200,799 bp) and *N. astigmatica* (125,042 bp) chloroplast genomes. Although gene content is well conserved among the three *Nephroselmis* chloroplast genomes, gene order is substantially scrambled. The *N. pyriformis* and *N. olivacea* chloroplast genomes are the most similar with respect to gene content, as both genomes share five of the seven genes that were previously found to be missing in *N. astigmatica* (*accD*, *cemA*, *ftsI*, *ftsW*, and *rnpB*) and as in the latter species, *rne* and *tilS* are lacking in *N*. *pyriformis*. The Long Single Copy (LSC) is 78,305 bp long, contains 74 protein-coding genes, a single non conserved ORF (ORF186a), *rnpB* and 25 tRNA. The Short Single Copy (SSC) is 19,693 bp long, contains 17 protein-coding genes, 2 non conserved ORFs (ORF120b and ORF160b) and 2 tRNA. The 6514-bp inverted repeat (IR) of *N. pyriformis* encodes 3 rRNA and 6 tRNA genes but no complete protein-coding gene, while the IRs of *N*. *olivacea* (46.1 kb) and *N*. *astigmatica* (13.7 kb) display several protein-coding genes in addition to rRNA and tRNA genes. As observed for *N. olivacea*, the *N. pyriformis* genome contains no introns. The putative protein encoded by *ftsH* is 3600 amino acid (aa) long in *N. pyriformis*, a size close to the corresponding *N. olivacea* sequence (3742 aa, GenBank: AAD54848) but much smaller than that of *N. astigmatica* (5,242 aa, GenBank: AID67672). The reverse situation was observed for *ycf1*, where the putative protein of *N. astigmatica* (AID67738, 1616 aa) is much longer than those of *N. pyriformis* (1074 aa) and *N. olivacea* (AAD54900, 956 aa). The putative proteins encoded by *ycf20* were found to be poorly conserved among the three *Nephroselmis* species (showing less than 35% sequence identity in pairwise Clustal Omega (Sievers et al. 2011) alignments), supporting the view that this gene could be a pseudo-gene in *Nephroselmis* (Lemieux et al. 2014).

A maximum likelihood phylogeny was inferred using RAxML version 8.0 (Stamatakis 2014) and the set of 71 genes that Lemieux et al. (2014) selected for their study of prasinophytes. Concatenated nucleotide sequences were aligned with MAFFT 7 (Katoh and Standley 2013) and variable regions in these alignments were trimmed with trimAl (Capella-Gutierrez et al. 2009). The phylogenic analysis was performed under the GTR + I + G model using *Chlorella vulgaris* (GenBank: NC_001865) as outgroup, with the best tree out of 100 being computed for 1,000 bootstrap replicates. The inferred tree identified the three *Nephroselmis* species within a strongly supported clade in which *N. pyriformis* is sister to *N. astigmatica* (Figure 1), a result that is in agreement with the 18S rRNA phylogeny reported by Lubiana et al. (2017). The presence of a gene-rich chloroplast genome in the basal *N. olivacea* taxa indicates that this feature is an ancestral characteristic of the genus and that several genes were lost in the marine lineage leading to *N. astigmatica*.

**Figure 1.**
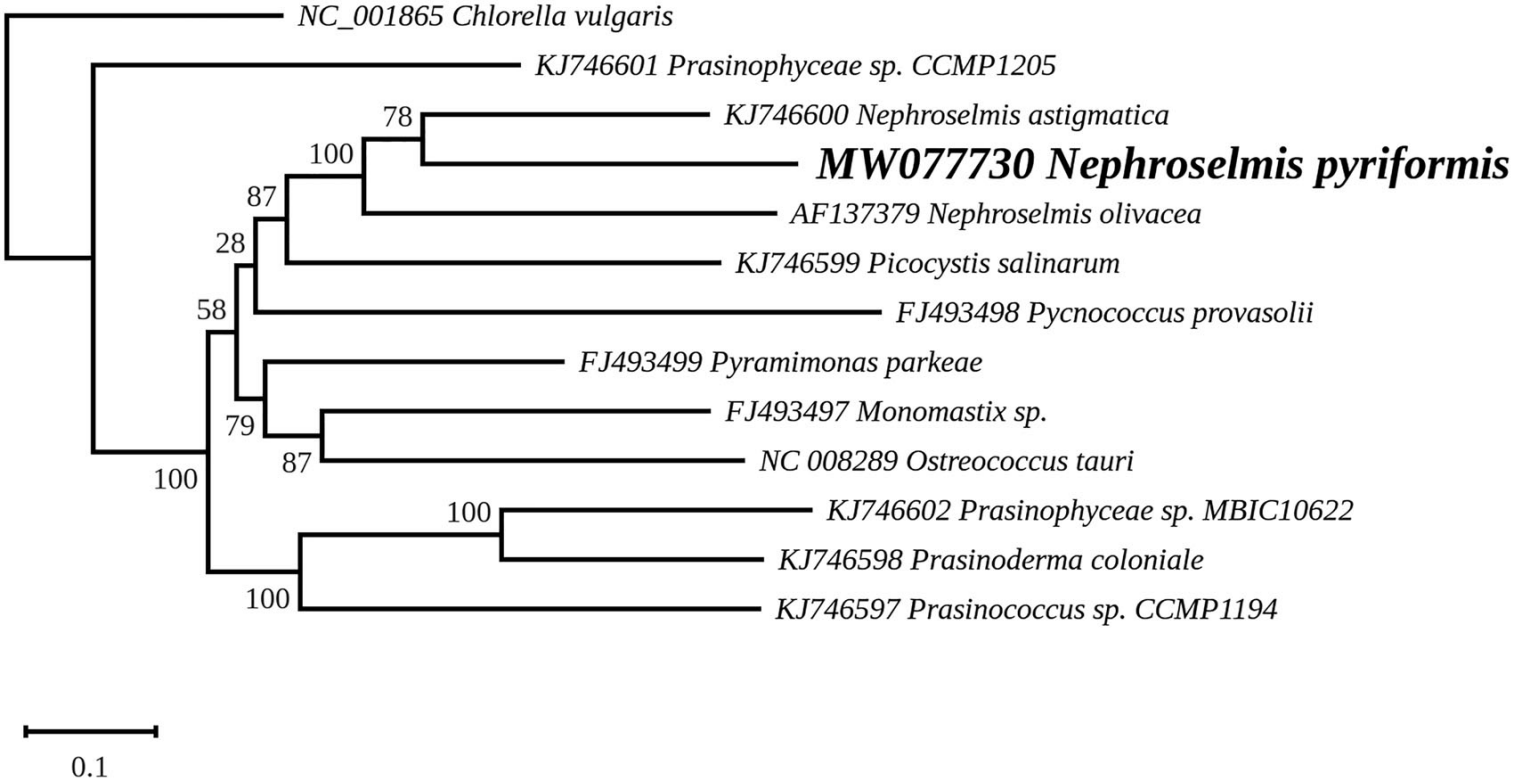
Maximum likelihood phylogeny obtained from 71 concatenated chloroplast protein-coding genes from Nephroselmis pyriformis and other selected Chlorophyta, with Chlorella vulgaris used as an outgroup. The best-scoring RAxML tree (log likelihood = –428,704.389139) is presented.

## Data Availability

The genome sequence data that support the findings of this study are openly available in GenBank of NCBI at (https://www.ncbi.nlm.nih.gov/) under the accession no. MW077730. The associated BioProject, SRA, and Bio-Sample numbers are PRJNA680225, SRR13108214, and SAMN16872557 respectively. The genome sequence data are also available on Zenodo with the following link: http://doi.org/10.5281/zenodo.4133421.
